# Unilateral Multicystic Dysplastic Kidney in a Fetus Associated With Parental Genetic and Environmental Risk Factors: A Case Report

**DOI:** 10.7759/cureus.102475

**Published:** 2026-01-28

**Authors:** Zuhayr Khan, Mahsum Jafri, Constantino G Lambroussis

**Affiliations:** 1 General Medicine, Lake Erie College of Osteopathic Medicine, Elmira, USA; 2 Internal Medicine, Lake Erie College of Osteopathic Medicine, Elmira, USA; 3 Osteopathic Medicine/Family Medicine, Lake Erie College of Osteopathic Medicine, Elmira, USA

**Keywords:** environmental lead exposure, fetal macrosomia, genetic and environmental risk factors, genetic risk factors, kidney ultrasound, multicystic dysplastic kidney, multifactorial etiology, nephrogenesis, ocd/anxiety disorders, unilateral renal cystic disease

## Abstract

Multicystic dysplastic kidney (MCDK) is a congenital renal anomaly identified on prenatal ultrasound. They often arise sporadically and unilaterally. Our case involves an isolated unilateral MCDK in a fetus born to a mother with generalized anxiety disorder (GAD), obsessive-compulsive disorder (OCD), and gastroesophageal reflux disease (GERD), with the father having chronic occupational lead exposure and a congenital disorder of glycosylation type 1A (PMM2-CDG). Our case highlights the multifactorial etiology of renal dysplasia and its potential role of glycosylation defects and environmental toxicity in abnormal kidney development. Contributions from genetic, environmental, and metabolic influences during nephrogenesis contribute to MCDK.

## Introduction

The development of multicystic dysplastic kidney (MCDK) is characterized by disorganized renal parenchyma that is replaced by multiple non-communicating cysts and fibrous stroma, which often results from disrupted ureteric bud-metanephric mesenchyme signaling during early nephrogenesis [1]. The estimated incidence of MCDK is one in 4,300 live births, with a male and left-sided kidney predominance [2]. It is known that most cases are unilateral and isolated; in addition, it is associated with excellent postnatal prognosis if the contralateral kidney is normal [3]. However, bilateral or syndromic forms do occur and can result in severe oligohydramnios and perinatal mortality [4].

Ultrasound (US) remains the cornerstone of the diagnosis of MCDK, which shows multiple non-communicating cysts of varying sizes with absent corticomedullary differentiation and loss of reniform shape [2,5]. The diagnostic accuracy of US in experienced centers approaches 90%-95% [4]. Adjunctive fetal magnetic resonance imaging (MRI) improves delineation of complex renal and extrarenal malformations, particularly in cases with limited visualization or oligohydramnios [6].

The origins of MCDK belong to the spectrum of congenital anomalies of the kidney and urinary tract, a group of systems influenced by both genetic and environmental mechanisms [1]. Studies have shown that mutations in genes regulating renal morphogenesis, such as HNF1B, PAX2, and uroplakins, have been implicated in MCDK [1]. Similarly, intrauterine exposure to extrinsic toxins such as heavy metals (lead, mercury, and chromium) may exacerbate oxidative and vascular injury during nephrogenesis [1]. The contribution of metabolic and glycosylation disorders, such as PMM2-CDG, to renal dysgenesis remains underrecognized but biologically plausible due to the importance of N-linked glycoproteins in nephron differentiation and ciliary signaling [1].

## Case presentation

A 25-year-old primigravida woman presented to the prenatal clinic at 21 weeks of gestation for a routine fetal anatomy US. She had an unremarkable early pregnancy course and was not taking any teratogenic medications. Her medical history was significant for obsessive-compulsive disorder (OCD), generalized anxiety disorder (GAD), and gastroesophageal reflux disease (GERD). These were managed conservatively without psychotropic or systemic pharmacotherapy at the time of this pregnancy. She also denied any tobacco, alcohol, or recreational drug use. The father, on the other hand, has a medical history notable for occupational lead exposure from industrial work and a known diagnosis of phosphomannomutase 2 congenital disorder of glycosylation type 1A (PMM2-CDG) identified during childhood. There was no known family history of congenital kidney or urinary tract malformations on either the maternal or paternal side of the family.

**Figure 1:**
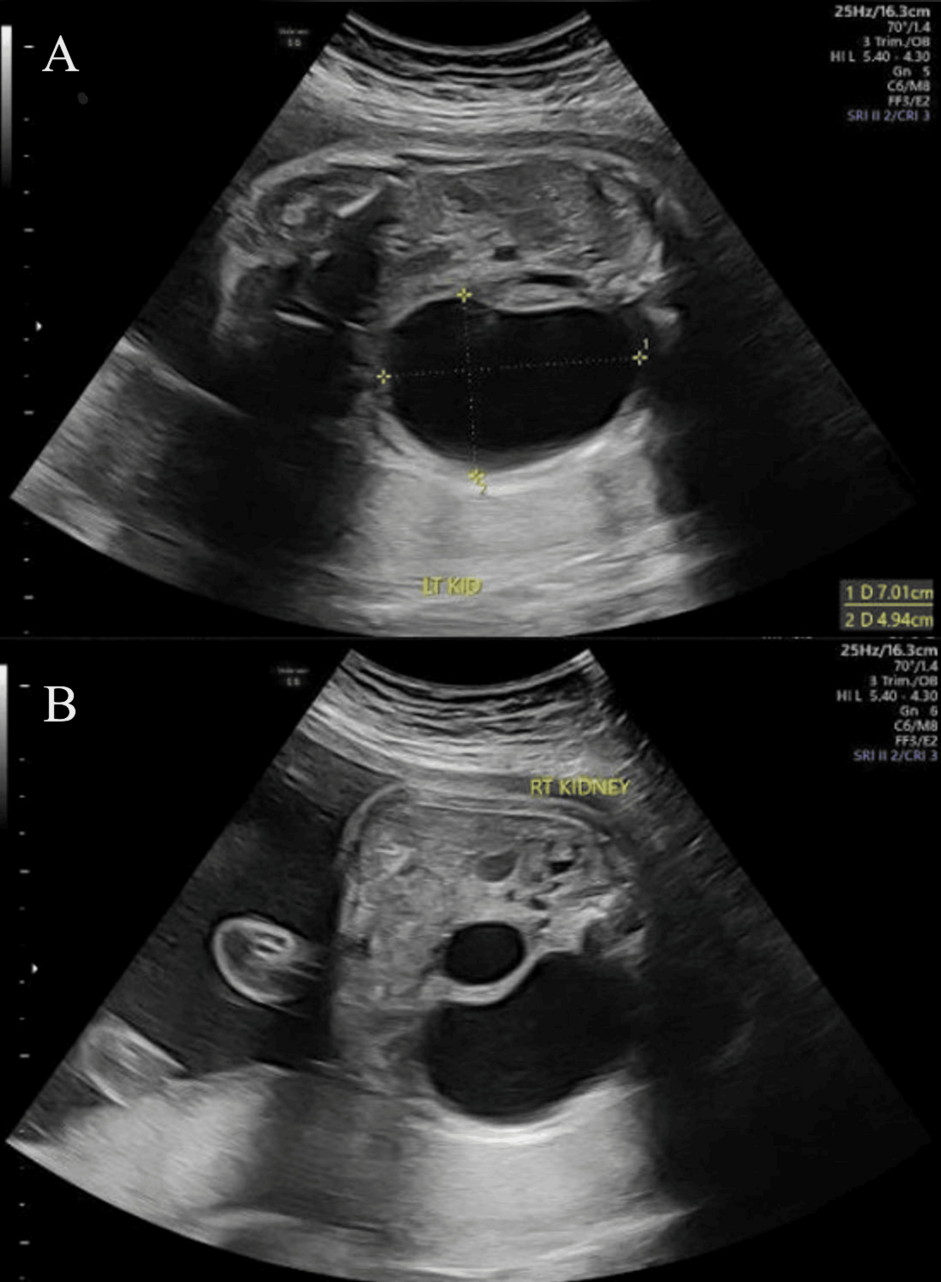
Prenatal abdominal ultrasound demonstrating the fetal kidneys in transverse view. (A) The left kidney (LT KID) appears enlarged with multiple non-communicating cystic spaces of varying sizes and loss of normal corticomedullary differentiation, with the largest measuring 7.01 × 4.94 cm. This is consistent with the diagnosis of MCDK. (B) The right kidney (RT KIDNEY) is shown in comparison with no abnormalities. MCDK: multicystic dysplastic kidney

During the routine mid-second-trimester scan, it was noted that there were some sort of abnormalities within the fetal kidneys, which prompted referral to the maternal-fetal medicine (MFM) clinic for further evaluation. At the MFM clinic, targeted ultrasonography confirmed an enlarged left renal fossa occupied by multiple, non-communicating cystic structures of varying sizes, with an average size of 6.7 cm (compared to a normal/unremarkable size of <2 cm). The normal reniform contour was lost, and no distinct renal sinus or corticomedullary differentiation was visualized. The right kidney appeared morphologically normal with an intact corticomedullary definition. Also, amniotic fluid volume was normal, and no additional fetal structural anomalies were detected. These findings confirmed a diagnosis of unilateral MCDK. These findings can be visualized in Figure 1, which shows the fetal kidneys at 29 weeks (third trimester) in the transverse abdominal view.

The patient and her partner were counseled regarding the diagnosis, potential outcomes, and management options. They were reassured that isolated unilateral MCDK typically carries a favorable prognosis due to the compensatory function of the contralateral kidney. Serial US every 1-2 weeks was recommended to monitor fetal growth, amniotic fluid levels, and the appearance of the contralateral kidney.

Follow-up imaging after the images seen in Figure 1 showed no significant progression in cyst size or number. It also showed renal dimensions that were still normal on the unaffected side, although the unaffected kidney did demonstrate mild compensatory hypertrophy. Signs suggestive of oligohydramnios, anhydramnios, or fetal distress were observed. During the third trimester at 32 weeks, the fetus developed macrosomia as the estimated fetal weight was above the 95th percentile. Given maternal discomfort and fetal size, an induction of labor was planned to be performed at 39 weeks’ gestation. The fetus’s weight at birth was borderline macrosomic at 3,800 g (~85th percentile), depending on an individual's preferred criteria of assessment.

The postnatal evaluation at birth included a renal US to assess the structure and function of both kidneys. In addition, there was an extensive workup to check for a non-functioning multicystic kidney and confirm compensatory hypertrophy of the contralateral side, urine output, serum creatinine, and electrolyte levels. Furthermore, postnatal follow-up appointments were regular for continued monitoring. In these follow-up appointments, serial USs were done to document involution of the dysplastic kidney and growth of the healthy kidney, along with regular assessments of renal function and overall development.

## Discussion

This case highlights how genetic susceptibility, environmental exposure, and metabolic stressors may converge to produce sporadic-appearing congenital anomalies of the kidney and urinary tract [1].

Pathophysiology and developmental considerations

Normal renal morphogenesis is dependent on reciprocal signaling between the ureteric bud and metanephric mesenchyme during weeks 5-10 of gestation [1,5]. If there is a disruption during this phase, most commonly through intrinsic mutations or extrinsic teratogenic effects, it can lead to cystic dysplasia and loss of nephron formation [1]. Thus, it essentially results from atresia of the ureteral bud system during embryogenesis [3]. Histologically, MCDK will exhibit primitive ducts, increased stroma, and cystic degeneration [1]. A somewhat alternative view is that disruption of normal nephrogenesis could, at least in part, be explained by impairment of fetal urine flow very early in fetal life; this is supported by the fact that an MCDK is generally attached to an “atretic,” non-patent ureter [4]. The hypothesis is supported by the association of urinary tract malformations in patients with renal dysplasia, including ureteral ectasia, ureterovesical stenosis, ureterocele, and urethral valves [7]. Moreover, the severity of the renal dysplasia is believed to depend on the timing of the obstructive defect relative to kidney morphogenesis [7].

The father in our case carried PMM2-CDG, an autosomal recessive disorder of N-linked glycosylation, which causes defective glycoprotein processing and has been associated with multisystemic involvement, including renal anomalies and tubular dysfunction [1]. This is significant as glycosylation influences renal morphogenesis by modifying receptor signaling, such as fibroblast growth factor and WNT pathways, which are critical to nephron branching [1]. In our case, paternal carrier status may have contributed to heterozygous or epigenetic modulation in the fetus, leading to a potential increase in susceptibility to dysplastic development, especially in conjunction with environmental stressors.

Environmental and metabolic contributors

Exposure to lead is known to cause oxidative stress, vascular injury, and altered calcium signaling in fetal tissues [1]. It has been shown that even at subclinical maternal levels, it has been linked to impaired renal growth and reduced nephron endowment in animal models [1]. Although direct causation cannot be confirmed in our case here, paternal lead exposure could contribute to germline DNA damage or epigenetic alterations transmitted to the embryo [1].

Another factor to consider is that the mother's metabolic and psychiatric conditions may have played an indirect role, as they can influence placental perfusion and oxidative balance [1]. Therefore, glucose (that is, a mother with diabetes) is recognized as a renal teratogen [8]. Although OCD and GAD themselves are unlikely to be the core issue in the development of MCKD in the fetus, chronic stress and altered cortisol levels have been hypothesized to affect fetal organogenesis through glucocorticoid receptor-mediated signaling [1]. Fetuses demonstrating abnormal growth patterns such as macrosomia may experience dysregulated insulin and insulin-like growth factor activity, which can influence renal growth trajectories and nephron maturation [9]. Thus, fetal macrosomia in our case further suggests possible subclinical maternal metabolic dysregulation or placental hyperfunction, which can stem indirectly from the mother's condition and ultimately alter the intrauterine environment during nephrogenesis [9].

Genetic-environmental interplay

The pathogenesis of MCDK shows the multifactorial behavior of congenital anomalies of the kidney and urinary tract, where genetic predisposition can interact with environmental insults [1]. Once a renal anomaly is detected prenatally, it is important to establish whether it is a part of a genetic complication [5]. This is seen in PMM2-CDG-related glycosylation defects, as it could weaken nephrogenic signaling, while simultaneous lead exposure and metabolic stress can amplify cellular injury [1]. Other genes implicated in the pathogenesis of MCDK include PEX26, ELN, HNF1B, ALG12, FRG1, FRG2, and CYP4A11 genes [10]. This additive effect may lead to localized renal dysplasia despite normal contralateral development [1]. It has been emphasized that such interactions likely underlie many sporadic cases of congenital anomalies of the kidney and urinary tract; therefore, it is speculated that future genetic screening may require multigene or pathway-level analyses [1].

Diagnostic modalities

The use of US remains the primary modality for prenatal detection of MCDK [5]. This is due to it being a reliable, non-invasive, and relatively inexpensive method for the measurement of kidney size [11]. The typical findings include multiple non-communicating cysts with the absence of renal sinus echoes and a non-visualized ureter [2,3]. US diagnosis for MCDK in utero has been undertaken for decades, and the sensitivity has ranged from 80% to 100% [4,12]. Therefore, it is also recommended that additional US follow-up should be implemented during pregnancy and that postnatal evaluation should be performed for possible associated anomalies [11].

Although US is the primary imaging for the prenatal diagnosis of fetal diseases [6], it has some limitations as it cannot accurately diagnose and analyze bilateral kidney anomalies with oligohydramnios, whereas MRI is not affected by these factors [6]. MRI is not hampered by the conditions of maternal obesity, oligohydramnios, and fetal pelvic bones [6]. The coronal and sagittal views on MRI can precisely identify the anatomy of bilateral kidneys and analyze the renal tissue and simultaneously detect central nervous and cardiovascular deformities on the same plane [6]. Overall, the use of MRI further refines assessment, especially in cases of high uncertainty, by providing multiplanar resolution and differentiating cystic dysplasia from hydronephrosis or polycystic kidney disease [6]. An alternative option if MRI is not available can include using a 3D US, as the examiner could reconstruct the region of interest and vividly reveal the extent and severity of MCDK, which may be missed or difficult to see during the traditional 2D US examination [12].

Prognosis and natural history

The prognosis for MCDK, when isolated and unilateral, is generally benign and not life-threatening [2,3]. It has been reported in most cases that there are favorable long-term outcomes with spontaneous involution, as well as compensatory hypertrophy of the contralateral kidney [3,11,13]. It was reported in a cohort of children with isolated unilateral MCDK diagnosed prenatally and found that there was complete involution of MCDK by 10 years in more than 50% of those affected [2,13]. There was no hypertension, malignancy, or significant proteinuria noted long-term. Surgical removal is indicated when the affected kidney is enlarging or if the mass effect from the MCDK impacts respiration or feeding in the neonate [2]. In contrast, bilateral MCDK is a rare condition with a poor prognosis, as infants with bilateral kidney disease often die during the neonatal period [14]. Bilateral disease, or association with extrarenal anomalies that are reported in up to 25%-40% of cases, significantly worsens prognosis [1,2]. Further solidifying the favorable outcomes of unilateral MCDK, it has been demonstrated that isolated hyperechogenic or cystic kidneys with normal amniotic fluid had over 70% normal renal outcomes postnatally [9].

## Conclusions

Through this interesting case and external sources, it was demonstrated that there is indeed a probable multifactorial pathogenesis of unilateral MCDK. The multifactorial attributes involved genetic susceptibility (PMM2-CDG carrier status), environmental toxicity (lead exposure), and other intrauterine metabolic factors (macrosomia, maternal stress). It is emphasized how early detection through routine US is key to diagnosis, as well as confirmation via postnatal imaging and multidisciplinary counseling, which remain key to the management of this condition. Overall, the recognition of underlying risk factors can enhance our understanding of congenital anomalies of the kidney and urinary tract and emphasize the importance of preconceptional and environmental health assessments to determine the risk of MCDK development.
